# A Novel Nanobody Targeting Middle East Respiratory Syndrome Coronavirus (MERS-CoV) Receptor-Binding Domain Has Potent Cross-Neutralizing Activity and Protective Efficacy against MERS-CoV

**DOI:** 10.1128/JVI.00837-18

**Published:** 2018-08-29

**Authors:** Guangyu Zhao, Lei He, Shihui Sun, Hongjie Qiu, Wanbo Tai, Jiawei Chen, Jiangfan Li, Yuehong Chen, Yan Guo, Yufei Wang, Jian Shang, Kaiyuan Ji, Ruiwen Fan, Enqi Du, Shibo Jiang, Fang Li, Lanying Du, Yusen Zhou

**Affiliations:** a State Key Laboratory of Pathogen and Biosecurity, Beijing Institute of Microbiology and Epidemiology, Beijing, China; b Lindsley F. Kimball Research Institute, New York Blood Center, New York, New York, USA; c Department of Veterinary and Biomedical Sciences, College of Veterinary Medicine, University of Minnesota, Saint Paul, Minnesota, USA; d ShanXi Agricultural University, Shanxi, China; e Northwest A&F University, Shaanxi, China; f Institute of Medical and Pharmaceutical Sciences, Zhengzhou University, Zhengzhou, China; Loyola University Medical Center

**Keywords:** MERS-CoV, spike protein, receptor-binding domain, nanobody, cross-neutralization, protective efficacy

## Abstract

The newly emerged Middle East respiratory syndrome coronavirus (MERS-CoV) continues to infect humans and camels, calling for efficient, cost-effective, and broad-spectrum strategies to control its spread. Nanobodies (Nbs) are single-domain antibodies derived from camelids and sharks and are potentially cost-effective antivirals with small size and great expression yield. In this study, we developed a novel neutralizing Nb (NbMS10) and its human-Fc-fused version (NbMS10-Fc), both of which target the MERS-CoV spike protein receptor-binding domain (RBD). We further tested their receptor-binding affinity, recognizing epitopes, cross-neutralizing activity, half-life, and efficacy against MERS-CoV infection. Both Nbs can be expressed in yeasts with high yield, bind to MERS-CoV RBD with high affinity, and block the binding of MERS-CoV RBD to the MERS-CoV receptor. The binding site of the Nbs on the RBD was mapped to be around residue Asp539, which is part of a conserved conformational epitope at the receptor-binding interface. NbMS10 and NbMS10-Fc maintained strong cross-neutralizing activity against divergent MERS-CoV strains isolated from humans and camels. Particularly, NbMS10-Fc had significantly extended half-life *in vivo*; a single-dose treatment of NbMS10-Fc exhibited high prophylactic and therapeutic efficacy by completely protecting humanized mice from lethal MERS-CoV challenge. Overall, this study proves the feasibility of producing cost-effective, potent, and broad-spectrum Nbs against MERS-CoV and has produced Nbs with great potentials as anti-MERS-CoV therapeutics.

**IMPORTANCE** Therapeutic development is critical for preventing and treating continual MERS-CoV infections in humans and camels. Because of their small size, nanobodies (Nbs) have advantages as antiviral therapeutics (e.g., high expression yield and robustness for storage and transportation) and also potential limitations (e.g., low antigen-binding affinity and fast renal clearance). Here, we have developed novel Nbs that specifically target the receptor-binding domain (RBD) of MERS-CoV spike protein. They bind to a conserved site on MERS-CoV RBD with high affinity, blocking RBD's binding to MERS-CoV receptor. Through engineering a C-terminal human Fc tag, the *in vivo* half-life of the Nbs is significantly extended. Moreover, the Nbs can potently cross-neutralize the infections of diverse MERS-CoV strains isolated from humans and camels. The Fc-tagged Nb also completely protects humanized mice from lethal MERS-CoV challenge. Taken together, our study has discovered novel Nbs that hold promise as potent, cost-effective, and broad-spectrum anti-MERS-CoV therapeutic agents.

## Abstract (precis)

Therapeutic development is critical for preventing and treating continual MERS-CoV infections in humans and camels. Because of their small size, nanobodies (Nbs) have advantages as antiviral therapeutics (e.g., high expression yield and robustness for storage and transportation) and also potential limitations (e.g., low antigen-binding affinity and fast renal clearance). Here, we have developed novel Nbs that specifically target the receptor-binding domain (RBD) of MERS-CoV spike protein. They bind to a conserved site on MERS-CoV RBD with high affinity, blocking RBD's binding to MERS-CoV receptor. Through engineering a C-terminal human Fc tag, the *in vivo* half-life of the Nbs is significantly extended. Moreover, the Nbs can potently cross-neutralize the infections of diverse MERS-CoV strains isolated from humans and camels. The Fc-tagged Nb also completely protects humanized mice from lethal MERS-CoV challenge. Taken together, our study has discovered novel Nbs that hold promise as potent, cost-effective, and broad-spectrum anti-MERS-CoV therapeutic agents.

## INTRODUCTION

Nanobodies (Nbs), also called camelid heavy-chain variable domains (VHHs), are single-domain nano-sized antibodies; they are derived from variable fragments of camelid or shark heavy chain-only antibodies (1, 2). Nbs contain four constant regions, named framework regions (FRs), and three connecting variable regions, called complementarity determining regions (CDRs). FRs are responsible for maintaining the structural integrity of Nbs, while CDRs directly bind to antigen epitopes (3). On the one hand, because of their nanometer size (∼2.5 nm by 4 nm) and single domain structure, Nbs have the following advantages as antiviral agents: they can be easily expressed for bulk production, they are robust for convenient storage and transportation, and they have good permeability in tissues (4–6). On the other hand, also because of their small size, Nbs have the following potential limitations as antiviral agents: they may have limited binding affinity for antigens and may be cleared from the body relatively quickly (the upper size limit of proteins for renal clearance is 60 kDa) (7, 8). Nevertheless, the use of Nbs as antiviral therapeutic agents is gaining more and more clinical acceptance, with the focus on overcoming their potential limitations (9–11).

Middle East respiratory syndrome coronavirus (MERS-CoV) was first identified in June 2012 (12) and continues to infect humans: it has led to at least 2,220 confirmed cases and 790 deaths (∼36% fatality rate) in 27 countries (http://www.who.int/emergencies/mers-cov/en/). Bats and dromedary camels are likely the natural reservoir and transmission hosts, respectively, for MERS-CoV. Whereas camel-to-human transmission of MERS-CoV has accounted for most of the human infections, human-to-human spread of MERS-CoV also occurs sporadically (13, 14). Currently, no therapeutic agents or vaccines have been approved for human use. Due to the continued threat of MERS-CoV, there is an urgent need to develop highly potent, cost-effective, and broad-spectrum anti-MERS-CoV therapeutics and vaccines with the potential for large-scale industrial production.

Therapeutic antibodies have been shown to be effective antiviral agents (15, 16). The receptor-binding domain (RBD) of MERS-CoV spike (S) protein is a prime target for therapeutic antibodies. The MERS-CoV S protein guides viral entry into host cells. It first binds to its host receptor dipeptidyl peptidase 4 (DPP4) through the RBD of its S1 subunit and then fuses viral and host membranes through its S2 subunit (15, 17–22). The RBD contains a receptor-binding motif (RBM) region (residues 484 to 567) that directly interacts with DPP4. We have previously shown that RBD-based vaccines are highly immunogenic and can induce the production of potent anti-MERS-CoV cross-neutralizing antibodies (23–27). Moreover, we have discovered several RBD-specific monoclonal antibodies (MAbs) with strong neutralizing activities against lethal MERS-CoV infections in human DPP4-transgenic (hDPP4-Tg) mice (15, 28, 29). These and some other RBD-targeting MAbs are currently being developed as anti-MERS-CoV therapeutics in experimental animal models (15, 30–36). However, the widespread use of conventional antibodies can be limited by their large size, high production costs, inconvenient storage and transportation, and poor pharmacokinetics (37), making Nbs attractive alternatives to traditional MAbs to treat MERS-CoV infections. Currently, it has not been shown whether MERS-CoV RBD can reliably trigger the production of Nbs, whether the produced Nbs can overcome the potential limitations (e.g., low binding affinity for the RBD and relatively short half-life in the body), or whether the produced Nbs can demonstrate sufficient therapeutic efficacy to warrant further development in clinical settings.

Here, after immunizing llama with recombinant MERS-CoV RBD protein, we generated a novel neutralizing Nb, NbMS10, and also constructed its human-Fc-fused version, NbMS10-Fc. We further investigated these Nbs for their RBD-binding capabilities, neutralization mechanisms, cross-neutralizing activity against divergent MERS-CoV strains, half-life, and protective efficacy against lethal MERS-CoV infection in an established hDPP4-Tg mouse model (38). This study reveals that efficacious, robust, and broad-spectrum Nbs can be produced to target MERS-CoV S protein RBD and that they hold great promise as potential anti-MERS-CoV therapeutics.

## RESULTS

### Identification and characterization of MERS-CoV-RBD-specific Nbs.

**FIG 1:**
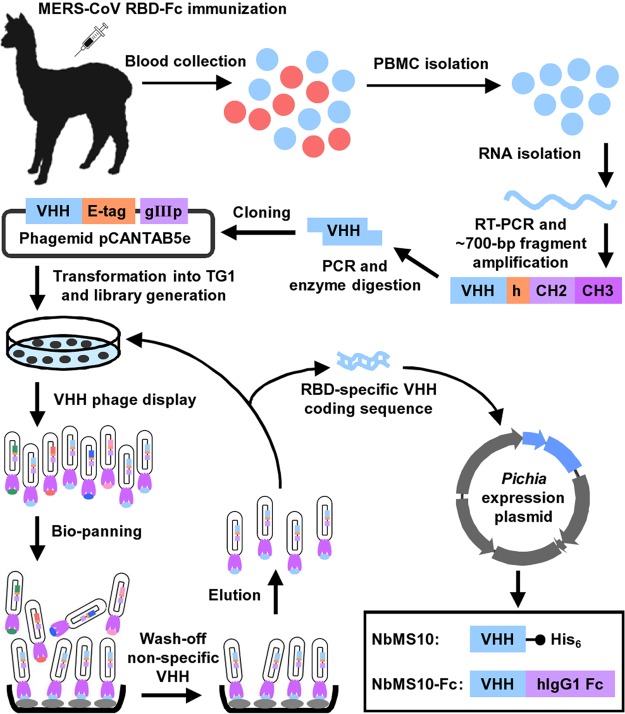
Schematic map for establishment of MERS-CoV Nb library and generation of NbMS10 and NbMS10-Fc Nbs. Blood was collected from MERS-CoV RBD-Fc protein-immunized alpaca after the last immunization to isolate PBMCs. RNA was then extracted to synthesize cDNA via RT-PCR. This was followed by PCR amplification of the N-terminal IgG heavy-chain fragment (∼700 bp), including the VHH gene, while the latter was used as the template to amplify the VHH gene fragment (∼300 to 450 bp). The VHH DNA sequence was further ligated into phagemid vector pCANTAB5e and transformed into E. coli TG1 competent cells to construct VHH library. VHH phage display was carried out to isolate RBD-specific clones. After four rounds of bio-panning, the RBD-specific VHH coding sequence was confirmed from the selected positive clones. The identified VHH coding gene containing a C-terminal His_6_ or human IgG1 Fc was inserted into Pichia pastoris yeast expression vector pPICZαA to construct NbMS10 and NbMS10-Fc, respectively, for further soluble expression and purification.

**FIG 2:**
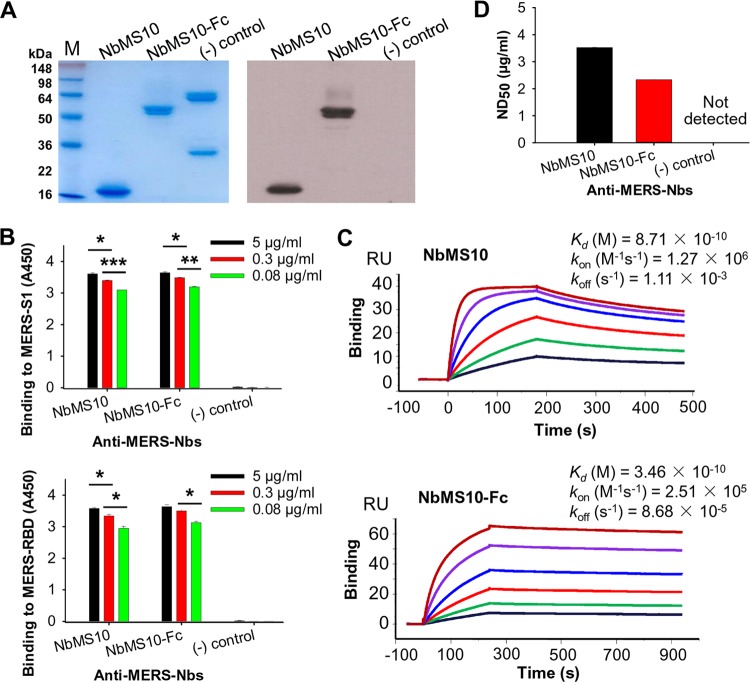
Characterization of MERS-CoV RBD-specific NbMS10 and NbMS10-Fc Nbs. (A) SDS-PAGE and Western blot analyses of purified NbMS10 and NbMS10-Fc. The Nbs were subjected to SDS-PAGE (left) or Western blotting (right), followed by detection using anti-llama antibody. The molecular weight marker (in kDa) is indicated on the left. (B) Detection of binding between NbMS10 or NbMS10-Fc and MERS-CoV S1 (MERS-S1) or RBD (MERS-RBD) protein by ELISA. The plates were coated with MERS-CoV S1-His or RBD-Fd protein (2 μg/ml), followed by sequential incubation with respective Nbs and goat anti-llama and HRP-conjugated anti-goat IgG antibodies. The data are presented as mean *A*_450_ values ± the standard deviation (SDs) (*n* = 2). Significant differences (*; **, and ***) are shown in the binding of Nbs to MERS-S1 or MERS-RBD at various concentrations. (C) The binding kinetics between NbMS10 or NbMS10-Fc and MERS-CoV RBD or S1 protein were measured by SPR. MERS-CoV RBD-Fc protein was used for binding to NbMS10 (containing a C-terminal His_6_), and S1-His protein was used for binding to NbMS10-Fc (containing a C-terminal human Fc). (D) Detection of NbMS10 and NbMS10-Fc neutralizing activity against MERS-CoV infection (EMC2012 strain) by a microneutralization assay. The Nb-MERS-CoV mixtures were incubated with Vero E6 cells and observed for the presence or absence of CPE. Neutralizing activity of Nbs was recorded as the concentration of Nbs in complete inhibition of MERS-CoV-induced CPE in at least 50% of the wells (ND_50_). The data are expressed as mean ND_50_ ± the SD (*n* = 3). The experiments were repeated twice, and similar results were obtained. The “(−) control” in panels A, B, and D refers to SARS-CoV 33G4 mouse MAb.

To construct the Nb (i.e., VHH) library, we immunized llama with recombinant MERS-CoV RBD (residues 377 to 588, EMC2012 strain) containing a C-terminal human IgG1 Fc tag (i.e., RBD-Fc) and isolated peripheral blood mononuclear cells (PBMCs) from the immunized llama. After four rounds of bio-panning and screening using MERS-CoV RBD-Fc, we isolated a positive clone with the highest binding affinity for the RBD. The gene encoding this RBD-specific Nb was subcloned into yeast expression vector to construct NbMS10 (which contains a C-terminal His_6_ tag) and NbMS10-Fc (which contains a C-terminal human IgG1 Fc tag) Nbs (Fig. 1). Both NbMS10 and NbMS10-Fc were expressed in yeast cells, secreted into the cell culture supernatants, and purified to homogeneity (Fig. 2A, left). The estimated molecular weights were about 16 kDa for NbMS10 and 50 kDa for NbMS10-Fc, since the latter formed a dimer. These MERS-CoV RBD-specific Nbs from llama, but not severe acute respiratory syndrome coronavirus (SARS-CoV) RBD-specific MAbs from mice, were recognized by anti-llama antibodies (Fig. 2A, right). Thus, the yeast-expressed Nbs maintained their native conformation and antigenicity.

To characterize their functions, we examined how the Nbs interact with MERS-CoV RBDs. First, we evaluated the binding between the Nbs and MERS-CoV RBD using ELISA. The result showed that both Nbs bound strongly to recombinant MERS-CoV RBD containing a C-terminal folden tag (RBD-Fd) and MERS-CoV S1 containing a C-terminal His_6_ tag (S1-His) in a dose-dependent manner (Fig. 2B). Second, we determined the binding affinity of the two Nbs for MERS-CoV RBD using surface plasmon resonance (SPR). The result showed that the *K_d_* between NbMS10 and RBD-Fc was 0.87 nM, whereas the *K_d_* between NbMS10-Fc and S1-His was 0.35 nM (Fig. 2C). Third, we carried out MERS-CoV neutralization assay. The result showed that the Nbs efficiently neutralized the infection of live MERS-CoV (EMC2012 strain) in Vero cells. The measured 50% neutralization doses (ND_50_) were 3.52 μg/ml for NbMS10 and 2.33 μg/ml for NbMS10-Fc (Fig. 2D). Taken together, the Nbs strongly bound to MERS-CoV RBD and neutralized MERS-CoV infection.

### Molecular mechanism underlying the neutralizing activities of Nbs.

**FIG 3:**
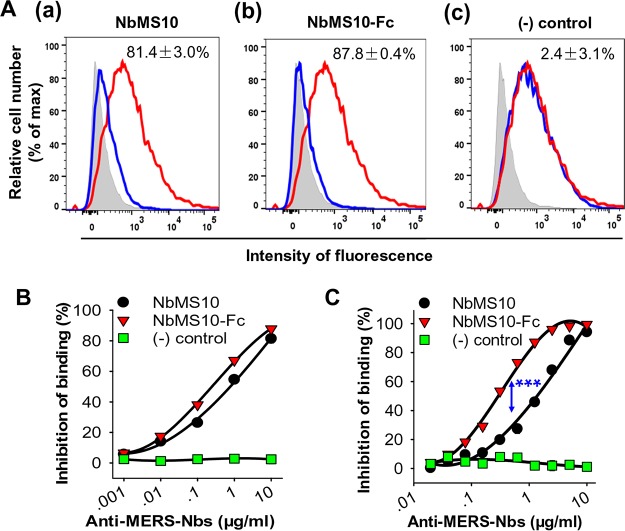
Determination of mechanisms of NbMS10 and NbMS10-Fc Nbs by flow cytometry and ELISA analyses. (A and B) Flow cytometry analysis of NbMS10 and NbMS10-Fc in inhibiting the binding between MERS-CoV RBD and cell-associated hDPP4 receptor. (A) Gray shading indicates the Huh-7 cell control. The red line indicates the binding of MERS-CoV RBD (i.e., RBD-Fc protein, 20 μg/ml) to Huh-7 cells. The blue line indicates NbMS10 (a) and NbMS10-Fc (b) Nbs (10 μg/ml) or the SARS-CoV 33G4 MAb control (c) inhibited RBD binding to Huh-7 cells. The percent inhibition values are shown in each graph. (B) NbMS10 and NbMS10-Fc demonstrated dose-dependent inhibition of the binding between MERS-CoV RBD and cell-associated hDPP4 in Huh-7 cells. The percent inhibition was calculated as the RBD-Huh-7 cell binding in the presence or absence of Nbs according to the following formula: (1 − RBD-Huh-7-Nb/RBD-Huh-7) × 100. (C) ELISA analysis of NbMS10 and NbMS10-Fc in inhibiting the binding between MERS-CoV RBD and soluble hDPP4 protein. The plates were coated with MERS-CoV RBD-Fc protein (2 μg/ml), followed by sequential incubation with serial dilutions of Nbs or hDPP4 protein (2 μg/ml), goat anti-hDPP4, and HRP-conjugated anti-goat IgG antibodies. The percent inhibition was calculated as the RBD-hDPP4 binding in the presence or absence of Nbs according to the following formula: (1 − RBD-hDPP4-Nb/RBD-hDPP4) × 100. A significant difference (***) occurred between NbMS10 and NbMS10-Fc in inhibition of RBD-hDPP4 binding. The “(−) control” in panels B and C refers to SARS-CoV 33G4 MAb. The data are presented as the mean percent inhibition ± the SD (*n* = 2). The experiments were repeated twice, and similar results were obtained.

To investigate the mechanism underlying the neutralizing activities of Nbs, we evaluated the competition between the Nbs and hDPP4 for the binding to MERS-CoV RBD. First, we carried out a flow cytometry assay where recombinant MERS-CoV RBD interacted with cell-surface-expressed DPP4 in the presence or absence of recombinant Nbs. The result showed that both Nbs significantly blocked the binding of RBD to cell-surface DPP4 in a dose-dependent manner (Fig. 3A and B). As a negative control, SARS-CoV-RBD-specific 33G4 MAb did not block the binding between MERS-CoV RBD and cell surface DPP4 (Fig. 3A and B). Second, we carried out an enzyme-linked immunosorbent assay (ELISA) where recombinant MERS-CoV RBD and recombinant hDPP4 interacted in the presence or absence of recombinant Nbs. The result showed that both Nbs, but not 33G4 MAb, blocked the binding between MERS-CoV RBD and DPP4 in a dose-dependent manner. Moreover, compared to NbMS10, NbMS10-Fc blocked the RBD-DPP4 binding more efficiently (Fig. 3C). These data reveal that the Nbs can compete with hDPP4 for the binding to MERS-CoV RBD, suggesting that the Nb-binding site and the DPP4-binding site overlap on the MERS-CoV RBD.

**FIG 4:**
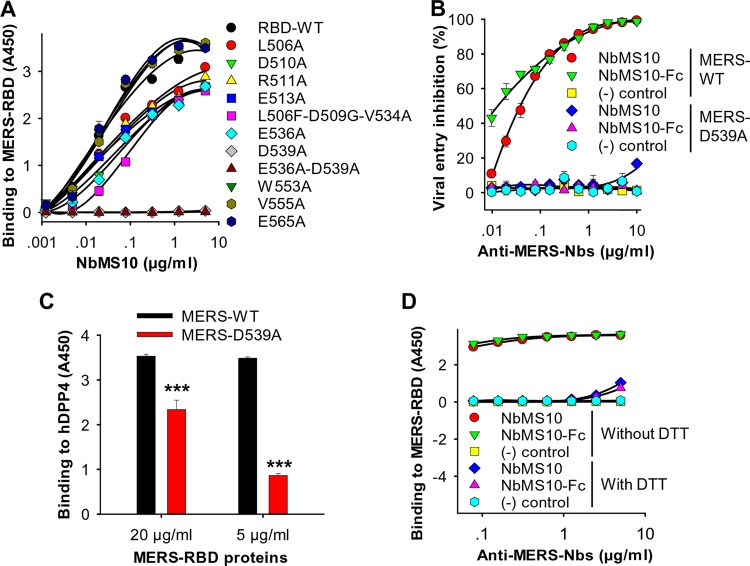
NbMS10 and NbMS10-Fc Nbs recognized conformational epitopes and mapping of Nb neutralizing epitope(s). (A) Mapping of the epitope of NbMS10 by ELISA. The plates were coated with RBD-Fc (RBD-WT) or respective mutant RBD proteins containing a C-terminal human Fc (2 μg/ml), followed by sequential incubation with serial dilutions of NbMS10 (containing a C-terminal His_6_), mouse anti-His and HRP-conjugated anti-mouse IgG antibodies. The data are presented as mean *A*_450_ values ± the SD (*n* = 3). (B) Inhibitory effect of NbMS10 and NbMS10-Fc against infection of MERS-CoV pseudoviruses with (MERS-D539A) or without (MERS-WT) D539A mutation. The data are presented as the mean percent inhibition ± the SD (*n* = 4). (C) Binding of MERS-CoV RBD with (MERS-D539A) or without (MERS-WT) D539A mutation to hDPP4 protein by ELISA. The data are presented as mean *A*_450_ values ± the SD (*n* = 4). A significant difference (***) occurred between MERS-WT and MERS-D539A in binding to hDPP4. (D) Detection of the binding between NbMS10 or NbMS10-Fc and MERS-CoV RBD by ELISA in the presence or absence of DTT. The plates were coated with RBD-Fd protein (2 μg/ml) and treated with or without DTT, followed by sequential incubation with serial dilutions of NbMS10 or NbMS10-Fc and goat anti-llama and HRP-conjugated anti-goat IgG antibodies. The data are presented as mean *A*_450_ values ± the SD (*n* = 2). The “(−) control” in panels B and D refers to SARS-CoV 33G4 MAb. The above-described experiments were repeated twice, and similar results were obtained.

To map the binding site of the Nbs on MERS-CoV RBD, we performed alanine scanning on the surface of MERS-CoV RBD and detected the binding of Nbs to the alanine-containing RBD mutants. The results showed that NbMS10 demonstrated tight binding to MERS-CoV RBD containing the single mutations L506A, D510A, R511A, E513A, E536A, W553A, V555A, and E565A and slightly reduced binding to RBD containing triple mutations L506F-D509G-V534A, suggesting that these RBD residues do not play significant roles in Nb binding. Instead, single mutation D539A and double mutations E536A-D539A on MERS-CoV RBD both ablated the binding of NbMS10 to the RBD (Fig. 4A), suggesting that RBD residue Asp539 plays an important role in Nb binding. We further investigated the role of Asp539 in Nb binding using the MERS-CoV pseudovirus entry assay. Neither NbMS10 nor NbMS10-Fc could neutralize the cell entry of MERS-CoV pseudovirus bearing the D539A mutation, again confirming that residue Asp539 is critical for Nb binding (Fig. 4B). To examine of the role of the D539A mutation in DPP4 binding, we carried out an ELISA to detect the binding between DPP4 and MERS-CoV RBD bearing the D539A mutation. The result showed that the D539A mutation significantly reduced the binding of the RBD to DPP4 (Fig. 4C). Overall, these results demonstrate that Nbs recognize the Asp539-containing epitope on MERS-CoV RBD and that this epitope also plays an important role in DPP4 binding. Therefore, the Nbs and DPP4 compete for the same region on MERS-CoV RBD, and mutations in this region can reduce the binding of both the Nbs and DPP4.

To investigate whether Nb-recognized epitopes on MERS-CoV RBD are conformational or linear, we detected the binding of Nbs to MERS-CoV RBD with its conformational structure disrupted. To this end, we treated MERS-CoV RBD with reducing agent dithiothreitol (DTT) to break the disulfide bonds in the protein, and performed an ELISA on the binding between Nbs and DTT-treated RBD. The result showed that neither NbMS10 nor NbMS10-Fc bound to the DTT-treated RBD (Fig. 4D). As a control, both Nbs bound to untreated RBD with high affinity. Thus, the Nbs recognize the conformational epitope on the RBD.

**FIG 5:**
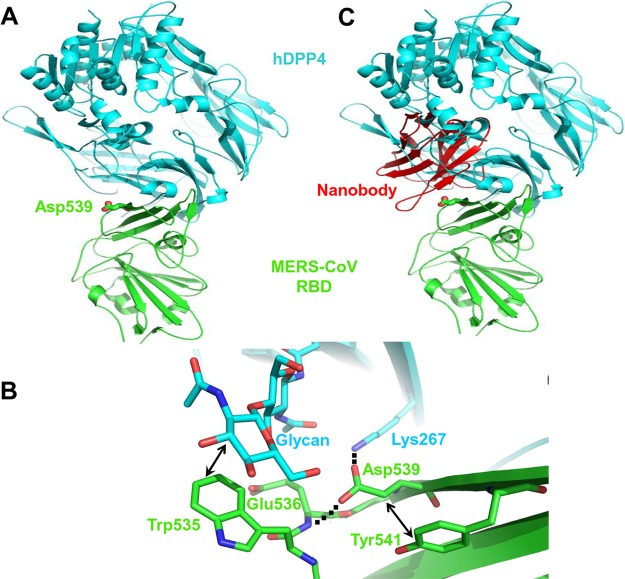
Proposed structural mechanisms for the neutralizing activity of NbMS10 and NbMS10-Fc Nbs. (A) Crystal structure of MERS-CoV RBD complexed with hDPP4 receptor (PDB 4KR0). MERS-CoV RBD is colored in green, and hDPP4 is colored in cyan. RBD residue Asp539, which is critical for the binding of the Nbs to the RBD, is shown in sticks. (B) Structural role of RBD residue Asp539 at the interface between MERS-CoV RBD and hDPP4 (PDB 4KR0). RBD residue Asp539 forms a critical salt bridge with DPP4 residue 267, a van der Waals interaction with RBD residue Tyr541, and a hydrogen bond with the main chain nitrogen of RBD residue Glu536. Near Asp539 is an N-linked glycan from DPP4 that forms strong and favorable van der Waals stacking with RBD residue Trp535. Dotted lines indicate hydrogen bonds, and arrows indicate van der Waals interactions. (C) Proposed structural mechanisms for the neutralizing activity of NbMS10 and NbMS10-Fc Nbs. The Nbs (colored in red) bind to the RBD epitope surrounding Asp539, disrupting the binding interactions between the RBD and DPP4 and physically blocking the binding of DPP4 to the RBD.

To understand the structural mechanism underlying the neutralizing activities of the Nbs, we examined the competitive interactions among the Nbs, DPP4, and MERS-CoV RBD using structural modeling (Fig. 5). In the absence of the Nbs, MERS-CoV RBD binds tightly to the DPP4 receptor, with D539 of RBD serving as a key residue at the binding interface (Fig. 5A). Here, RBD residue D539 forms a critical salt bridge with DPP4, and it interacts with the surrounding key RBD residues via van der Waals contacts and hydrogen bonds (Fig. 5B), enabling RBD and DPP4 to maintain strong binding interactions. The Nbs bind tightly to the RBD in the same D539-containing region, abolishing the binding between RBD and DPP4 (Fig. 5C).

### Cross-neutralizing activity of Nbs against divergent MERS-CoV strains.

To investigate the cross-neutralizing activity of Nbs against divergent MERS-CoV isolates, we performed MERS-CoV pseudovirus entry assay in the presence of the Nbs where the pseudoviruses encode the S gene of various MERS-CoV isolates from different countries (Saudi Arabia, Qatar, and South Korea), hosts (human and camels), and time periods (2012 to 2015). These MERS-CoV strains all contain mutations in their RBDs. The results showed that both Nbs potently neutralized the cell entry of all of the MERS-CoV pseudoviruses, with the ND_50_ values ranging from 0.003 to 0.979 μg/ml (for NbMS10) and from 0.003 to 0.067 μg/ml (for NbMS10-Fc) (Table 1). Therefore, although the Nbs were developed using the RBD from one MERS-CoV strain (EMC2012), they have broad-spectrum cross-neutralizing activity against existing MERS-CoV strains, as well as potentially future emerging MERS-CoV strains.

**TABLE 1:** Cross-neutralizing activity of MERS-CoV RBD-specific Nbs against divergent strains of MERS-CoV^*a*^

Accession no.	Isolate yr	Host	Region	RBD mutation(s)^*b*^	ND_50_ (μg/ml)^*c*^	
					NbMS10	NbMS10-Fc
AFS88936	2012	Human	Saudi Arabia		0.046	0.047
AGV08379	2012	Human	Saudi Arabia	D509G	0.067	0.067
AGV08584	2012	Human	Saudi Arabia	V534A	0.979	0.026
AHI48528	2013	Human	Saudi Arabia	A431P, A482V	0.121	0.005
AHI48733	2013	Human	Saudi Arabia	A434V	0.049	0.003
AHC74088	2013	Human	Qatar	S460F	0.031	0.005
AHY22545	2013	Camel	Saudi Arabia	K400N	0.088	0.014
AHY22555	2013	Camel	Saudi Arabia	A520S	0.040	0.044
AID55090	2014	Human	Saudi Arabia	T424I	0.044	0.005
AID55087	2014	Human	Saudi Arabia	Q522H	0.156	0.005
ALB08322	2015	Human	South Korea	D510G	0.003	0.005
ALB08289	2015	Human	South Korea	I529T	0.004	0.011

a A pseudovirus-based neutralization assay was performed to evaluate the cross-neutralizing activity of Nbs against divergent MERS-CoV isolates. Pseudotyped MERS-CoV mutants were generated containing the corresponding mutations in the RBD of S protein of MERS-CoV representative isolates from 2012 to 2015.

b RBD residues mutated in the S protein of the respective pseudotyped MERS-CoV mutants are indicated. The pseudotyped MERS-CoV expressing S protein of the EMC2012 strain (accession no. AFS88936) was considered to be the prototype pseudovirus.

c The ND_50_ was determined as the 50% neutralization dose using a pseudotyped MERS-CoV neutralization assay.

### *In vivo* half-life of Nbs.

**FIG 6:**
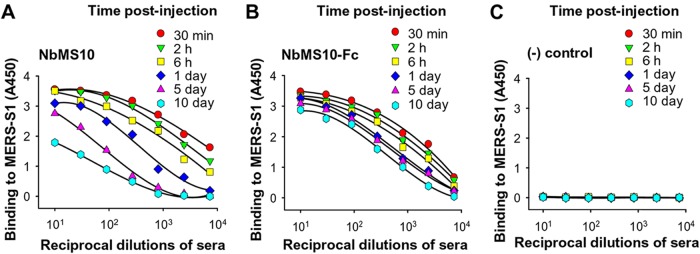
Detection of half-lives of Nbs in C57BL/6 mice. Sera were collected from mice injected with NbMS10 (A), NbMS10-Fc (B), or PBS control (C) at the indicated time points and then tested by ELISA for binding with the MERS-CoV S1 protein. The plates were coated with S1-His protein (2 μg/ml), and the data are presented as mean *A*_450_ values ± the SD of mice (*n* = 5) in each group.

To evaluate the *in vivo* half-life of the Nbs, we injected the Nbs into mice, collected the sera from the mice after different time intervals, and measured the binding between the sera and recombinant MERS-CoV S1 using ELISA. The results showed that the sera collected from NbMS10-injected mice gradually lost their binding affinity for MERS-CoV S1, and completely lost their binding for MERS-CoV S1 10 days postinjection (Fig. 6A). In comparison, NbMS10-Fc demonstrated stable binding for recombinant MERS-CoV S1 at 10 days postinjection (Fig. 6B). As a control experiment, sera collected from PBS-injected mice showed no binding for recombinant MERS-CoV S1 (Fig. 6C). Thus, compared to monomeric Nb, Fc-fused Nb has a significantly extended *in vivo* half-life likely due to its dimeric structure, which increases the molecular weight of Nb from 16 to 50 kDa and hence may slow down its renal clearance.

### Prophylactic and therapeutic efficacy of Nb in transgenic mice.

**FIG 7:**
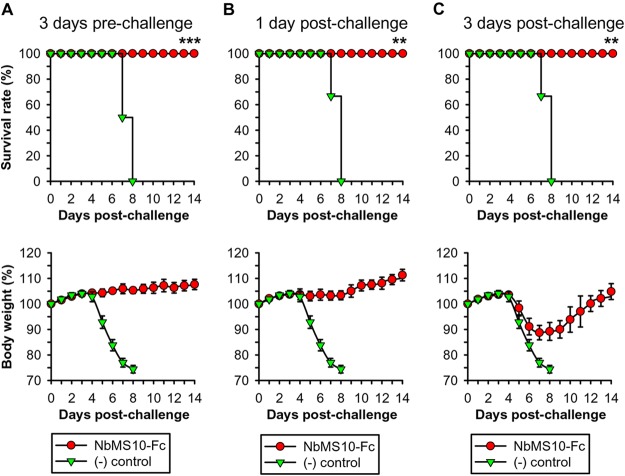
Evaluation of prophylactic and therapeutic efficacy of NbMS10-Fc in hDPP4-Tg mice. The hDPP4-Tg mice were treated with NbMS10-Fc or Trastuzumab “(−) control” (10 mg/kg) 3 days preinfection (A) or 1 day (B) and 3 days (C) postinfection with the MERS-CoV (EMC2012 strain, 10^5.3^ TCID_50_). Virus-challenged mice were monitored for 14 days to evaluate survival rate (above) and body weight changes (below). The body weight data are presented as means ± the SD of mice in each group (*n* = 6). Significant differences (** and ***) are indicated between the NbMS10-Fc and control groups.

Because MERS-CoV does not infect wild-type mice, we previously developed hDPP4-Tg mice (38) as the susceptible animal model for MERS-CoV research. To evaluate the prophylactic efficacy of NbMS10-Fc, mice were injected with a single dose of NbMS10-Fc 3 days before they were infected with a lethal dose of MERS-CoV and were subsequently monitored for their weight and survival. Trastuzumab, an antibody used for treating breast cancer, was used as a control. The result showed that after MERS-CoV infection, mice treated with NbMS10-Fc had a 100% survival rate (Fig. 7A, above) and steady weight (Fig. 7A, below). In comparison, mice treated with trastuzumab all died on day 8 postinfection, and their weight also sharply decreased starting from day 4 postinfection (Fig. 7A). To evaluate the therapeutic efficacy of NbMS10-Fc, mice were first infected with MERS-CoV and then treated with single-dose NbMS10-Fc either 1 or 3 days postinfection. The result showed that mice treated with NbMS10-Fc on day 1 postinfection had a 100% survival rate and steady weight (Fig. 7B). In addition, mice treated with NbMS10-Fc on day 3 postinfection also had a 100% survival rate (Fig. 7C, above); although their weights first decreased on day 5 postinfection, it rebounded on day 7 postinfection (Fig. 7C, below). In comparison, mice receiving trastuzumab all died on day 8 after infection, and their weights continuously decreased (Fig. 7B and C). Overall, NbMS10-Fc has potent prophylactic and therapeutic efficacy in protecting susceptible animal models against lethal MERS-CoV challenge.

## DISCUSSION

MERS-CoV continues to infect humans with a high fatality rate. Because camels likely serve as the transmission hosts for MERS-CoV and also because humans have contact with camels, the constant and continuing transmissions of MERS-CoV from camels to humans make it difficult to eradicate MERS-CoV from the human population. Thus, efficacious, cost-effective, and broad-spectrum anti-MERS-CoV therapeutic agents are needed to prevent and treat MERS-CoV infections in both humans and camels. Nbs have been gaining acceptance as antiviral agents because of their small size, good tissue permeability, and cost-effective production, storage, and transportation. However, their small size may also lead to relative low antigen-binding affinity and quick clearance from the host body. In this study, we have developed a novel MERS-CoV-targeting Nb, NbMS10, and its Fc-fused version, NbMS10-Fc, both of which demonstrate great promise as anti-MERS-CoV therapeutic agents.

NbMS10 and NbMS10-Fc present superior characteristics common to other Nbs. They target the MERS-CoV RBD, which plays an essential role in cell entry of MERS-CoV by binding to its receptor hDPP4. Both Nbs can be expressed in yeast cells with high purity and yields and are soluble in solutions. All of these properties suggest cost-effective production, easy storage, and convenient transportation of these Nbs in potential commercial applications.

The MERS-CoV RBD-targeting Nbs developed also demonstrate good qualities comparable to previously reported MERS-CoV RBD-specific conventional IgGs. First, the Nbs bind to MERS-CoV RBD with high affinities. The *K_d_* values for NbMS10 and NbMS10-Fc to bind MERS-CoV RBD were 8.71 × 10^−10^ M and 3.46 × 10^−10^ M, respectively. The *K_d_* values for RBD-targeting conventional IgGs to bind MERS-CoV RBD range from 7.12 × 10^−8^ M to 4.47 × 10^−11^ M (29, 35, 36). Moreover, the ND_50_ values for NbMS10 and NbMS10-Fc to neutralize MERS-CoV (EMC2012 strain) infection in cultured cells were 3.52 and 2.33 μg/ml, respectively. The ND_50_ values for RBD-specific conventional IgGs to neutralize various MERS-CoV strains ranged from micrograms/ml to nanograms/ml (30, 32, 35, 39, 40). Thus, the Nbs developed in this study and conventional IgGs reported previously have comparable MERS-CoV RBD-binding affinities and MERS-CoV-neutralizing activities. Structural comparisons of conventional IgGs and Nbs have shown that the antigen-binding site of IgGs consists of paired heavy-chain and light-chain variable (VH-VL) domains, whereas Nbs lack the light chain and hence cannot form the paired VH-VL domains (8, 41). Instead, Nbs have an extended CDR3 region (>16 amino acid residues), longer than that of the VHs of conventional IgGs (average length 12 amino acid residues) (42–44). Moreover, the Nbs developed here contain a 22-amino-acid CDR3; the extended CDR3 enables the Nbs to bind to the antigens with higher affinity (37). Furthermore, although the single-domain Nb (i.e., NbMS10) is small and can be cleared from the serum relatively quickly, the Fc-fused Nb (i.e., NbMS10-Fc) with relatively increased size demonstrates extended *in vivo* half-life. Therefore, the potential short half-life of Nbs can be overcome by adding the appropriate tag to the Nbs to increase their half-life. Overall, the present study has shown the feasibility of overcoming the potential limitations of Nbs.

**FIG 8:**
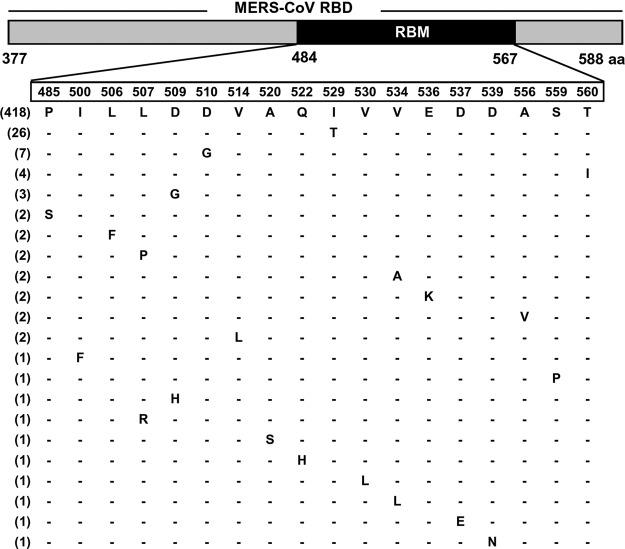
Conservation of residue D539 at the RBD of MERS-CoV S protein. Schematic structure of RBD and mutations of amino acid (aa) residues at the RBM of RBD among natural MERS-CoV isolates. A total of 482 RBM sequences (residues 484 to 567) derived from natural MERS-CoV isolates were aligned, and residues with natural mutations are shown. Residues in the rectangle frame show the RBM consensus, and the positions of corresponding residues are illustrated. The numbers on the left indicate the counts of MERS-CoV isolates with the identical sequence in the analyzed region.

The MERS-CoV RBD-targeting Nbs potently neutralize MERS-CoV entry into host cells. The *K_d_* values between the Nbs and MERS-CoV RBD are significantly lower than that between MERS-CoV RBD and hDPP4 receptor. As a result, the Nbs can outcompete hDPP4 for the binding of MERS-CoV RBD, thereby blocking the binding of MERS-CoV to DPP4, as well as MERS-CoV entry into host cells. It is worth noting that the RBD on the MERS-CoV S trimer frequently undergoes conformational changes, switching between a lying down, receptor-inaccessible conformation and a standing-up, receptor-accessible conformation. Hence, in the context of the virus particles where the RBD is part of the S protein, the Nbs would need to bind the RBD when the RBD is in the standing-up conformation (45). Importantly, the Nbs demonstrate strong cross-neutralizing activities against various MERS-CoV strains isolated from different hosts (humans and camels) and from different time points during MERS-CoV circulation in humans (from years 2012 to 2015). NbMS10 had a relatively high ND_50_ against the AGV08584/2012 strain containing a V534A mutation, which is consistent with the slightly reduced binding affinity between NbMS10 and MERS-CoV RBD containing the V534A mutation (Fig. 4A). The broad neutralizing spectrum of the Nbs results from the binding site of the Nbs on MERS-CoV RBD, which is located in the Asp539-containing region that plays a critical role in DPP4 binding. Interestingly, several MERS-CoV RBD-specific conventional IgGs also bind to the same epitope (39, 46), suggesting that this region is a hot spot for immune recognition. Although mutations in this region can eliminate the binding of the Nbs to MERS-CoV RBD and hence lead to viral immune evasion, they also reduce the binding of MERS-CoV RBD to receptor DPP4 and hence decrease the efficiency of viral entry. Thus, viral immune evasion from the inhibition of the Nbs through mutations can be costly to MERS-CoV itself. Indeed, residue Asp539 in S protein RBD is highly conserved in almost all of the natural MERS-CoV strains published to date (Fig. 8). Therefore, the MERS-CoV-specific Nbs can potentially be developed into broad-spectrum anti-MERS-CoV therapeutic agents. Despite the above analysis, this study did not examine all possible mutations in the Nb-binding region (since the atomic structures of MERS-CoV RBD complexed with the Nbs are still unknown), and thus it is possible that future escape mutations may occur to residues that this study did not cover. In that case, a combination of the current Nbs and other antibodies targeting other S regions or various RBD epitopes may be helpful in battling the emergence of immune escape MERS-CoV strains.

In sum, the MERS-CoV-specific Nbs developed in the present study possess superior qualities common to all Nbs such as their small size and cost-effective production. They also overcome potential limitations of other Nbs by maintaining a high binding affinity for their target MERS-CoV RBD and an optimized half-life. Moreover, they recognize a functionally important region on MERS-CoV RBD, rendering viral immune evasion costly and at the same time making themselves good candidates as broad-spectrum anti-MERS-CoV therapeutics. We have confirmed the effectiveness of the Nbs by showing that the Fc-fused Nb completely protected animal models from lethal MERS-CoV challenge. Thus, the Nbs can potentially be used in both humans and camels to prevent and treat MERS-CoV infections in either of these hosts and also block the camel-to-human transmission of MERS-CoV. Overall, our study proves the feasibility of developing highly effective Nbs as anti-MERS-CoV therapeutic agents and points out strategies to preserve the advantages of Nbs, as well as to overcome the potential limitations of Nbs.

## MATERIALS AND METHODS

### Ethics statement.

The animal studies were carried out in strict accordance with the recommendations in the Guide for the Care and Use of Laboratory Animals of the State Key Laboratory of Pathogen and Biosecurity at the Beijing Institute of Microbiology and Epidemiology of China and the National Institutes of Health (NIH). The animal protocols were approved by the IACUC of the State Key Laboratory of Pathogen and Biosecurity, Beijing Institute of Microbiology and Epidemiology (permit BIME 2015-0024) and by the Committee on the Ethics of Animal Experiments of the New York Blood Center (approval 194.18).

### Construction of VHH library and screening for MERS-CoV-RBD-specific Nbs.

Construction of the Nb (i.e., VHH) library and screening of MERS-CoV-RBD-specific Nbs were performed as previously described (47). Briefly, male and female alpacas (llama pacos, 1 year) were subcutaneously immunized with recombinant RBD-Fc (260 μg/alpaca) (48) plus Freund complete adjuvant, and boosted three times with the same immunogen plus Freund incomplete adjuvant (InvivoGen). Blood was collected 10 days after the last immunization, and then PBMCs were isolated using Ficoll-Paque gradient centrifugation (GE Healthcare). Total RNA was extracted with TRIzol reagent (Invitrogen). cDNA was synthesized by reverse transcription-PCR (RT-PCR) using a TransScript cDNA Synthesis SuperMix (TransGen Biotech, China), followed by PCR amplification of the N-terminal IgG heavy-chain fragment (∼700 bp), using the forward primer VHH-L-F (5′-GGTGGTCCTGGCTGC-3′) and the reverse primer CH2-R (5′-GGTACGTGCTGTTGAACTGTTCC-3′). The VHH gene (∼300 to 450 bp) was further amplified using the above DNA fragment as the template and the forward primer VHH-FR1-D-F (5′-TTTCTATTACTAGGCCCAGCCGGCCGAGTCTGGAGGRRGCTTGGTGCA-3′) and the reverse primer VHH-FR4-D-R (5′-AAACCGTTGGCCATAATGGCCTGAGGAGACGRTGACSTSGGTC-3′) (the SfiI restriction site is underlined). The SfiI-digested VHH DNA fragment was then inserted into phagemid vector pCANTAB5e (Bio-View Shine Biotechnology, China) to construct the VHH phage display library (49). Phage particles were analyzed by ELISA using recombinant MERS-CoV RBD-Fc and Fc of human IgG1 proteins as the positive and negative target proteins, respectively, to screen for RBD-specific Nbs. After four rounds of bio-panning, one of five positive clones, CAb10, with the highest binding to MERS-CoV RBD, was selected for further analyses (Fig. 1).

### Expression of MERS-CoV-RBD-specific Nbs in yeast cells.

NbMS10 and NbMS10-Fc Nbs containing a C-terminal His_6_ and Fc of human IgG1, respectively, were constructed based on the aforementioned CAb10 VHH. The DNA sequences encoding NbAb10 and NbAb10-Fc were synthesized (GenScript) and inserted into the Pichia pastoris secretory expression vector, pPICZαA (Invitrogen) (Fig. 1). The recombinant NbMS10 and NbMS-Fc were expressed in Pichia pastoris GS115 cells and purified using a Ni-NTA column (for NbMS10; GE Healthcare) and a protein A Sepharose 4 Fast Flow column (for NbMS10-Fc; GE Healthcare), respectively.

### SDS-PAGE and Western blotting.

The purified anti-MERS-CoV-RBD Nbs were analyzed using SDS-PAGE and Western blotting (23, 48). Briefly, Nbs (3 μg) were loaded onto 10% Tris-glycine SDS-PAGE gels and stained using Coomassie brilliant blue or transferred to nitrocellulose membranes. After being blocked overnight at 4°C with 5% nonfat milk/phosphate-buffered saline–Tween 20 (5% PBST), the membranes were incubated sequentially with goat anti-llama IgG (1:3,000; Abcam) and horseradish peroxidase (HRP)-conjugated anti-goat IgG (1:1,000; R&D Systems) antibodies for 1 h at room temperature and then with ECL Western blot substrate reagents. Finally, the membranes were visualized using Amersham Hyperfilm (GE Healthcare). A SARS-CoV-RBD-specific MAb, 33G4 (50), was used as a control.

### ELISA.

ELISA was performed to detect the binding between Nbs and MERS-CoV S1 or RBD proteins (23, 51). Briefly, ELISA plates were coated overnight at 4°C, respectively, with recombinant MERS-CoV S1-His (48), RBD-Fc (48), RBD-Fd (51), or one of the mutant RBDs containing a C-terminal human Fc tag (28). After being blocked with 2% PBST for 2 h at 37°C, the plates were further incubated sequentially with serially diluted Nbs (containing a C-terminal His_6_ or Fc tag), either goat anti-llama (1:5,000) or mouse anti-His (1:3,000) antibody (Sigma) and either HRP-conjugated anti-goat IgG (1:3,000) or HRP-conjugated anti-mouse IgG (1:5,000) antibody (GE Healthcare) for 1 h at 37°C. ELISA substrate (3,3′,5,5′-tetramethylbenzidine [TMB]; Invitrogen) was added to the plates, and the reactions were stopped with 1 N H_2_SO_4_. The absorbance at 450 nm (*A*_450_) was measured using a Tecan Infinite 200 Pro microplate reader (Tecan).

To detect the binding between Nbs and denatured MERS-CoV RBD protein, ELISA plates were coated with RBD-Fd protein (2 μg/ml) overnight at 4°C and then sequentially incubated with DTT (10 mM) and iodoacetamide (50 mM) (Sigma) for 1 h at 37°C (28). After three washes using PBST, ELISA was performed as described above.

Inhibition of the binding between MERS-CoV RBD and hDPP4 proteins by Nbs was performed using ELISA as described above, except that recombinant hDPP4 protein (2 μg/ml; R&D Systems), and serially diluted Nbs were added simultaneously to the RBD-Fc-coated plates. The binding between RBD and DPP4 was detected using goat anti-hDPP4 antibody (1:1,000; R&D Systems) and HRP-conjugated anti-goat IgG (1:3,000). The percent inhibition was calculated based on the *A*_450_ values of RBD-hDPP4 binding in the presence or absence of Nbs. SARS-CoV 33G4 MAb was used as a negative control to Nbs.

### Surface plasmon resonance.

The binding between Nbs and MERS-CoV S1 or RBD protein was detected using a BiacoreS200 instrument (GE Healthcare) as previously described (29). Briefly, recombinant Fc-fused MERS-CoV RBD-Fc protein or NbMS10-Fc Nb (5 μg/ml) was captured using a Sensor Chip protein A (GE Healthcare), and recombinant His_6_-tagged MERS-CoV S1-His protein or NbMS10 Nb at various concentrations was flown over the chip surface in a running buffer containing 10 mM HEPES (pH 7.4), 150 mM NaCl, 3 mM EDTA, and 0.05% surfactant P20. The sensorgram was analyzed using Biacore S200 software, and the data were fitted to a 1:1 binding model.

### Flow cytometry.

This assay was performed to detect the inhibition of the binding between MERS-CoV RBD and cell surface hDPP4 by Nbs (28). Briefly, Huh-7 cells expressing hDPP4 were incubated with MERS-CoV RBD-Fc protein (20 μg/ml) for 30 min at room temperature in the absence or presence of Nbs at various concentrations. Cells were incubated with fluorescein isothiocyanate-labeled anti-human IgG antibody (1:50, Sigma) for 30 min and then analyzed by flow cytometry. The percent inhibition was calculated based on the fluorescence intensity of RBD-Huh-7 cell binding in the presence or absence of Nbs.

### MERS pseudovirus neutralization assay.

Neutralization of MERS pseudovirus entry by Nbs was performed as previously described (23, 52). Briefly, 293T cells were cotransfected with a plasmid encoding Env-defective, luciferase-expressing HIV-1 genome (pNL4-3.luc.RE) and a plasmid encoding MERS-CoV S protein. The MERS pseudoviruses were harvested from supernatants at 72 h posttransfection and then incubated with Nbs at 37°C for 1 h before being added to Huh-7 cells. After 72 h, the cells were lysed in cell lysis buffer (Promega), incubated with luciferase substrate (Promega), and assayed for relative luciferase activity using Tecan Infinite 200 Pro Luminator (Tecan). The ND_50_ of the Nbs was calculated as previously described (53).

### MERS-CoV microneutralization assay.

Neutralization of MERS-CoV infection by Nbs was performed as previously described (28, 54). Briefly, MERS-CoV (EMC2012 strain) at an amount equal to 100 median tissue culture infective doses (TCID_50_) was incubated with Nbs at different concentrations for 1 h at 37°C. The Nb-virus mixture was then incubated with Vero E6 cells for 72 h at 37°C in the presence of 5% CO_2_. The cytopathic effect (CPE) was observed daily. The neutralizing activity of Nbs was reported as the ND_50_. The Reed-Muench method was used to calculate the ND_50_ value for each Nb (55).

### Measurement of half-life of Nbs.

Male and female C57BL/6 mice (6 to 8 weeks old) were intravenously injected with Nbs (50 μg in 200 μl per mouse) into the tail vein. Sera were collected at different time points (30 min, 2 h, 6 h, 1 day, 5 days, and 10 days postinjection). The concentrations of Nbs in the sera were detected by ELISA, as described above. Briefly, MERS-CoV S1-His protein (2 μg/ml) was used to coat ELISA plates, and then sera, goat anti-llama antibodies (1:5,000), and HRP-conjugated anti-goat IgG antibodies (1:3,000) were sequentially added for ELISA reactions.

### Evaluation of protective efficacy of NbMS10-Fc Nb.

The prophylactic and therapeutic efficacy of NbMS10-Fc was evaluated in hDPP4-Tg mice as previously described (29). Briefly, male and female mice (8 to 10 weeks old) were intraperitoneally anesthetized with sodium pentobarbital (5 mg/kg of body weight) before being intranasally inoculated with lethal dose of MERS-CoV (EMC2012 strain, 10^5.3^ TCID_50_) in 20 μl of Dulbecco modified Eagle medium. Either 3 days preinfection or 1 or 3 days postinfection, the mice were intraperitoneally injected with NbMS10-Fc (10 mg/kg). Trastuzumab MAb was used as a control to the Nb. The infected mice were observed daily for 14 days, and their body weights and survivals were recorded.

### Statistical analysis.

Statistical analysis was performed using GraphPad Prism version 5.01. To compare the binding of Nbs to MERS-CoV S1 or RBD protein, as well as the RBDs with or without D539A mutation to hDPP4 receptor, a two-tailed Student *t* test was used. One-way analysis of variance was used to compare the inhibition of Nbs to RBD-hDPP4 binding. Statistical significance between survival curves was analyzed using Kaplan-Meier survival analysis with a log-rank test. *P* values lower than 0.05 were considered statistically significant. In the figures, “*,” “**,” and “***” indicate *P* < 0.05, *P* < 0.01, and *P* < 0.001, respectively.

### Data availability.

All data needed to evaluate the conclusions presented here are included. Additional data related to this study may be requested from the authors.
